# Out-of-pocket expenditure experienced by couples seeking In Vitro Fertilization (IVF) services at tertiary care facilities in India

**DOI:** 10.1371/journal.pone.0351080

**Published:** 2026-07-15

**Authors:** Prerana Patil, Akshita Vikani, Deepshikha Sharma, Oshima Sachin, Biju Soman, Bhavani Shankara Bagepally, Renu Tanwar, Vanita Suri, V. Radha, M. Anitha, P. Ramesh, Deepti Shrivastava, Sukhpreet Patel, Shankar Prinja, Beena Joshi1, IVF Project Team

**Affiliations:** 1 Department of Operational & Implementation Research, Indian Council of Medical Research-National Institute of Research in Reproductive and Child Health, Mumbai, Maharashtra, India; 2 Department of Community Medicine and School of Public Health, Post Graduate Institute of Medical Education and Research, Chandigarh, Chandigarh, India; 3 Health Technology Assessment in India, Department of Health Research, New Delhi, Delhi, India; 4 Achutha Menon Centre for Health Science Studies, Sree Chitra Tirunal Institute of Medical Sciences and Technology, Trivandrum, Kerala, India; 5 Health Technology Assessment Resource Hub, Indian Council of Medical Research, National Institute of Epidemiology, Chennai, Tamil Nadu, India; 6 Department of Obstetrics and Gynaecology, IVF & Reproductive Biology Centre, Maulana Azad Medical College, New Delhi, Delhi, India; 7 IVF Centre, Post Graduate Institute of Medical Education and Research, Chandigarh, Chandigarh, India; 8 Department of Reproductive Medicine & Surgery, Sri Ramachandra Institute of Higher Education & Research, Chennai, Tamil Nadu, India; 9 Department of Reproductive Medicine, SAT Hospital, Government Medical College, Trivandrum, Kerala, India; 10 Obstetrics & Gynaecology, Jawaharlal Nehru Medical College, Datta Meghe Institute of Medical Sciences, Wardha, Maharashtra, India; King Saud University / Zagazig University, EGYPT

## Abstract

**Objective:**

Out-of-pocket expenditure (OOPE) incurred by infertile couples seeking In Vitro Fertilization (IVF) treatment in selected public and private hospitals in India.

**Design:**

Cross-sectional observational study.

**Setting:**

Two private and three public tertiary care hospitals across varied geographical regions of India that provide IVF services.

**Participants:**

A total of 148 couples undergoing IVF were enrolled via semi-purposive consecutive sampling over a six-month period after providing informed written consent.

**Primary outcome measures:**

Using validated structured tool, direct health costs (procedures, drugs, diagnostics), non-medical costs (travel, food, lodging), and indirect costs (loss of wages) were explored. Catastrophic health expenditure (CHE) and predictors of OOPE were also assessed.

**Results:**

The mean OOPE per IVF cycle was ₹1,10,104 (±₹75,503) [~USD 1,321(±906)] in public hospitals and ₹2,30,668 (±₹1,09,556) [~USD 2,768 (±1315)] in private hospitals. The majority of expenses were on drugs and diagnostics. Approximately 30% of couples experienced CHE, with drug costs contributing 55% of total expenditure. Only 5% had insurance coverage, with an average cover of ₹1,00,625 (±35,500) [~USD 1,208 (± 426)]. Over half (58%) relied on external financial support, mostly from friends and family.Couples without insurance had significantly higher OOPE than those with coverage (p=0.029). Higher household income was also linked to increased OOPE (p=0.001). Couples undergoing treatment at private facilities had 7.7 (CI: 2.5-23.15) times higher odds of experiencing CHE compared to public facilities. Lack of insurance coverage was associated with higher odds of CHE, although not significant.

**Conclusions:**

Couples undergoing IVF in public and private hospitals face substantial financial burdens, often resulting in CHE. This justifies the need to improve access to comprehensive infertility services, including IVF, through public health facilities to meet the demand for this emerging health problem.

## Introduction

According to the WHO, approximately 8–12% of couples worldwide experience primary infertility, which translates to an estimated 50–80 million couples globally, whereas the overall prevalence of primary infertility in India ranges from 3.9-16.8% [1]. Several factors contribute to this, including cultural stigmas surrounding infertility, lack of awareness about reproductive health, and limited access to fertility treatments [2]. Several different factors may cause infertility in either the male or female reproductive system. In India, the demographic landscape is marked by a paradoxical trend: while the overall population continues to grow, the Total Fertility Rate (TFR) has been steadily declining over recent decades. According to the National Family Health Survey (NFHS-5, 2019–21), the national TFR has dropped to 2.0 children per woman, which is at or below the replacement level in many states, reflecting significant progress in family planning and reproductive health interventions [3]. However, an important yet underexplored dimension in these surveys is the issue of infertility, which remains largely unaddressed in mainstream demographic data and policy frameworks.

The National Health Mission (NHM), through its Reproductive, Maternal, Newborn, Child, and Adolescent Health (RMNCH) program, comprehensively addresses various aspects of reproductive health but does not explicitly include infertility prevention or treatment. Similarly, the Pradhan Mantri Jan Arogya Yojana (PMJAY), a flagship public health insurance scheme, currently does not cover infertility-related treatments, leaving affected individuals to bear the high out-of-pocket expenditures (OOPE) associated with infertility care [4].

This presents a critical challenge, as India appears to be at a demographic crossroads characterized by simultaneous population growth and rising infertility rates. While government initiatives and public health programs have traditionally focused on curbing high fertility rates to manage population growth, there is an increasing need to recognize and respond to the growing prevalence of infertility. Infertility not only affects individuals’ reproductive choices and well-being but also has broader social and economic implications. This has become a major health challenge in India, with approximately 8% of infertile couples requiring intensive medical intervention through advanced Assisted Reproductive Technologies (ART) like In Vitro Fertilization (IVF) or Intracytoplasmic Sperm Injection (ICSI) [5].

IVF is an assisted reproductive technology in which an egg is fused with the sperm outside the body (In Vitro). The process of IVF involves ovarian stimulation, egg retrieval, sperm collection, fertilization in the laboratory, and embryo culture, followed by embryo transfer [6]. However, these advanced treatments can be expensive and are often beyond the reach of many Indians. Additionally, successful ART procedures demand substantial technical expertise and infrastructure. The clinical pregnancy rate per IVF transfer ranges from 32% to 35%, on basis of several factors, including the patient’s age, the underlying cause of infertility, and their history of treatment [7]. Among available reproductive treatments, IVF is widely regarded as one of the most effective options, with success rates that can be relatively high compared to other methods. Infertility is one of the important reproductive health problems increasingly faced by couples in India; but its integration in public health planning remains limited.

Utilization of IVF in India has been increasing significantly due to rising indications for treatment, coupled with awareness, changing societal norms, and advancements in reproductive technologies [8]. The demand for IVF services has surged, with many couples seeking assisted reproductive technologies due to factors such as delayed childbirth, lifestyle changes, and increased incidence of infertility. According to the National ART & Surrogacy Registry, India has 1454 registered ART clinics [9]. Estimates suggest that around 2-2.5 lakh IVF cycles are performed annually in India, making it one of the leading countries for assisted reproductive treatments [10]. The growing demand is fulfilled by an expanding private sector that provides these services. While the ART regulations govern these private providers, their prices remain unregulated [11].There is a lack of scientific data to suggest the cost of providing these services in India. While IVF is available in many urban centers, accessibility remains a challenge in rural areas due to costs and lack of awareness.

In India, over 50% of health expenditures are covered by out-of-pocket payments, placing a significant financial strain on households [12]. Out-of-pocket expenditures (OOPEs) can be catastrophic or impoverishing, constituting 58.7% of the total national health expenditure in India [13]. A 2020 economic survey estimated that raising public health spending to 3% of gross domestic product (GDP) could reduce out-of-pocket expenditures (OOPE) to 30% of total health expenditure [14].

According to the World Bank, the average Indian annual household income is 3.6 lakh (in 2022–2024), while the average cost of a single IVF cycle ranges from INR 1,50,000 to INR 4,0,0000. [~USD 1800 to USD 4800] [15]. Many couples may require multiple cycles to achieve a successful pregnancy, significantly increasing their total expenditure [16]. For many families, the out-of-pocket expenses associated with IVF can lead to catastrophic health expenditures, particularly for low- and middle-income households. The lack of insurance coverage for fertility treatment exacerbates this issue, forcing couples to pay entirely out of their own pockets [17].

There have been limited studies have explored the OOPE incurred by couples undergoing IVF in India, the determinants and the coping strategies used by couples [18]. This manuscript describes the spectrum of OOPE experienced by couples seeking IVF treatment at both private and public health facilities and the determinants.

Given the rising prevalence of infertility alongside ongoing demographic changes, there is a pressing need for policymakers to explicitly include infertility prevention and treatment within public health frameworks. Equally important is addressing the significant financial burden faced by couples, by reducing out-of-pocket expenditures and ensuring affordable access to assisted reproductive technologies like IVF. Hence at the behest of National Mission, a study was commissioned a to assess the expenditure incurred by couples seeking IVF treatment.

## Methodology

Study design and study site: This study was conducted at five tertiary hospitals in India, providing IVF services, including two private hospitals and three public hospitals. The selected sites were spread across various geographical regions (North, south and west). Ethical approvals were obtained from all the study sites and the coordinating centre (ICMR-NIRRCH).

**Ethical approval:** The study was conducted in accordance with ethical standards.

1. ICMR-NIRRCH Ethics Studies for Clinical studies 513/2022.
2. Sri Ramchandra Institute of Higher Education and Research-Institutional Ethics Committee IEC-NI/22/DEC/85/134
3. Post Graduate Institute of Medical Education and Research-Institutional Ethical Board: PGI/IEC/2023/000050. Approval Board: No. PGI/ICRC/2023/606
4. Human Ethics Committee, Medical College, Thiruvananthapuram- HEC NO: 09/17/2023/MCT
5. Maulana Azad Medical College-Institutional Ethics Committee- F.1/IEC/MAMC/96/02/2023/ No.360 dated 02/06/2023
6. Datta Meghe Institute of Higher Education and Research Institute- Ethics committee-DMIMS (DU)/IEC/2023/11 dated 03 Feb 2023

### Sampling & sample size

The sample size was calculated using estimates from a previous Indian study on IVF-related expenditure (Mukherjee et al., 2012; https://doi.org/10.4103/0974-1208.101014). The mean out-of-pocket expenditure (OOPE) per IVF cycle was reported as INR 48,173 (~USD 585) in 2012. After adjusting for inflation to 2022 levels, the mean cost was estimated at INR 83,798. As the standard deviation (SD) was not reported in the reference study, it was conservatively assumed to be equal to the mean (SD = INR 83,798) (~USD 1006). Assuming a small to moderate effect size of 0.25, a two-tailed α error of 0.05, and a power of 80%, the estimated sample size was 128 participants. To account for potential data incompleteness or dropouts, the final sample size was increased to 130 couples, equally distributed across the five selected health facilities.

148 IVF participants utilizing the services at selected sites were enrolled via semi-purposive consecutive sampling over six months and data were collected for one IVF cycle. Couples undergoing IVF at the facility, regardless of their diagnosis or treatment outcome, and those who were willing to participate in the study and provide informed consent, were included. Individuals who were unwilling to participate or did not provide consent were excluded.

### Data collection

A tested structured questionnaire was used to elicit socio-demographic details, self-reported illness, medical history, household consumption expenditure, along with healthcare expenditure at each stage of the IVF cycle from the Pre-IVF cycle, Follicle Study, Oocyte retrieval/Ovum Pickup (OPU) to Fresh or Frozen Embryo Transfer (ET). (The structured questionnaire has been added to the supplementary file)

Couples were enrolled at the embryo transfer stage, and retrospective data on costs incurred for a single IVF cycle were obtained during face-to-face interviews with couples visiting the facility for treatment. In order to limit recall bias, data were obtained during the ongoing IVF treatment process, specifically at the embryo transfer phase. Expenditure details were cross-checked with available documentation, and retrospective reporting was restricted to a short, well-defined period within the same treatment cycle. The reference period for cost reporting was restricted to a single IVF cycle, typically completed within approximately two months. This relatively short recall window substantially reduced the likelihood of recall errors. In cases where documentary evidence was available, which applied to the majority of participants, expenses were directly validated against bills and medical records. When documents were not available, detailed probing during interviews was used to improve the accuracy of self-reported amounts. All authors had access to identifiable participant information during data collection, and all data were de-identified before analysis. The data were collected from public hospitals at SAT, Trivandrum, from 25/07/2023 to 15/09/2023; at MAMC, Delhi, from 11/07/2023 to 31/08/2023; and at PGIMER, Chandigarh, from 07/07/2023 to 29/01/2024. Data was collected from private hospitals at SRIHER, Chennai, from 06/06/2023 to 03/08/2023, and from AVBRH, Wardha, from 05/06/2023 to 06/09/2023.

### Data analysis

The entered data was cleaned and analyzed using IBM SPSS version 20 and MS Excel software.

The collected data was analyzed to assess direct health costs, direct non-medical costs, and Indirect costs. The Direct Health Costs captured the expenditure incurred for registration, consultations, USG, laboratory tests, medicines, along with procedure charges. Procedure charges included any diagnostic procedure along with any laparoscopic procedure in the Pre-IVF stage, oocytes retrieval and embryo transfer (Fresh or Frozen) stage. It also included hospital admission charges, semen donation, semen donor charges (if applicable) and cryopreservation charges (if applicable). All the charges related to Ovarian Hyperstimulation Syndrome or any other complications were considered and the proportion of these components within the total direct health costs was analyzed.

Under non-medical costs, total travel costs, total food costs, and total lodging costs for all the visits during all four stages of the IVF cycle were considered including that of the bystander. The share of different components in the total non-medical cost was also analyzed.

Indirect costs, focusing on the total loss of wages due to paid work missed by the husband, wife, and any other accompanying person was analyzed.

All the above data was collated to arrive at Out-of-pocket expenditure estimates. The annual household expenditure data was also collected to estimate the catastrophic health expenditure incurred by couples for one IVF cycle. The threshold for catastrophic health expenditure was taken as 40% of annual household consumption expenditure without the expenditure incurred on food, as recommended by the WHO [19].

Linear and logistic regression analysis was undertaken to assess key factors contributing to out-of-pocket expenditure (OOPE) and catastrophic health expenditure (CHE) referring to key variables like service utilization (public/private), insurance coverage, per capita household income, type and causes of infertility, duration of infertility treatment, age of the female, receipt of financial support, use of donor sperm and other relevant socio-demographic characteristics. All potential confounding factors were controlled for, and reported adjusted differences in mean out-of-pocket expenditures and adjusted odds ratios.

## Results

Each site could enroll about 30 couples, totaling 148 in 6 months. More than half sought IVF for primary infertility and women were above 30 years of age. Table 1 below depicts the demographic profile of the 148 couples undergoing IVF at selected study sites.

**Table 1:** Background characteristics (IVF).

N = 148		Male	Female
Age in years (Mean ±SD)		37 (±5)	33 (±5)
Education		Illiterate-0% Primary-6.8% Secondary-17.6% Higher Secondary – 17.6% Diploma-5.4% Graduate-37.8% Post graduate-14.2% PhD-0.7%	Illiterate-0% Priamry-5.4% Secondary-13.5% Higher Secondary-16.2% Diploma-2% Graduate-39.2% Post graduate-22.3% PhD-1.4%
Employment		100%	21%
Married since (Mean ±SD) years		8 (±4)	
Years of undergoing treatment (mean ±SD)	infertility	6 (±4)	
Number of clinics visited for the treatment till date (mean ±SD)		4 (±3)	
Type of infertility		Primary: 59%	Secondary:41%
Family history of infertility		Male- 7%	Female-6%
Tobacco consumption		Male-18%	Female-1%
Alcohol consumption		Male- 19%	Female-2%
Underwent IUI No. of IUI cycles (Mean ± SD) Donor sperm used in the current cycle		49% 3 (±2) 6%	
Annual household income (Median, IQR)		Rs. 3,60,000 (240000,600000) USD 4320 (USD 2880–7200)	
Co-morbid conditions:History of hypothyroidism: History of Diabetes:		Male: 0% Male: 13%	Female- 30% Female-0%

Only 5% of the enrolled couples had insurance coverage for IVF treatment. The median annual insurance installment was Rs. 6,000 (IQR: 0, 17,750) [USD 72 (IQR: 0, 213)], and an average of Rs. 1,00,625 (±35,500) [USD 1,208 (±426)] of the treatment expense was covered by insurance. The majority of the participants (58%) had obtained financial support of which 60% had borrowed from friends and family, while others had taken bank loans or gold pledges.

Female factor infertility was the most prevalent, accounting for 72% of cases, while male factor and combined male and female factor infertility each accounted for 14%. Oligospermia and tubal factor infertility were the leading causes of infertility among male and female participants undergoing IVF treatment.

Table 2 depicts that fixed hospital charges were the same for all participants undergoing one IVF cycle at the particular facility, while non-medical and indirect costs varied. The average cost paid as fixed hospital charges was the mandatory payment done by the participants for one IVF cycle to the hospital.

**Table 2:** Out-of-pocket Expenditure incurred by couples undergoing IVF (presented in INR and US dollars).

Study Sites	Direct Health Cost (Investigations, procedure, drugs, etc.)		Non-Medical Cost (Food, travel & lodging) Mean ±SD	Indirect health cost (Loss of wage- Husband & wife) Mean ±SD	Average OOPE Mean ±SD
	Fixed hospital charge	Cost incurred in addition to fixed hospital charges Mean ±SD			
**PGIMER,** **Chandigarh** **(Public)**	Rs.23,000	Rs. 55,529 (±18,450)	Rs.11,055 (± 10,212)	Rs.3,614* (± 1,787)	**Rs.90,616** **(± 22,485)**
	$ 276	$ 666 (±221)	$ 133 (±123)	$ 43 (±21)	$ **1087** **(±270)**
**MAMC,** **Delhi** **(Public)**	–	Rs.45,479 (± 16,093)	Rs.5,778 (±2,516)	Rs.3,618 (± 3,174)	**Rs. 54,876** **(± 21,782)**
		$ 546 (±193)	$ 69 (±30)	$ 43 (±38)	$ **659** (±**261)**
**SAT,** **Trivandrum** **(Public)**	Rs.40,000	Rs. 1,13,067 (± 83,784)	Rs. 18,802 (± 13,619)	Rs.11,653 (± 10,465)	**Rs. 1,83,522** **(± 84,952)**
	$ 480	$ 1,357(±1005)	$ 226 (±163)	$ 140 (±126)	$ **2,202 (**±**1,019)**
**AVBRH,** **Wardha** **(Private)**	Rs.95,000	Rs. 33,020 (± 25,153)	Rs.3,507 (± 3,305)	Rs.4,050* (± 3,025)	**Rs. 1,32,337** **(± 26,932)**
	$ 1,140	$ 396 (±302)	$ 42 (±40)	$ 49 (±36)	$ **1,588** (±**323)**
**SRIHER,** **Chennai** **(Private)**	Rs. 1,97,450	Rs. 1,15,879 (± 59,308)	Rs.11,806 (± 6981)	Rs.6,104* (± 5,075)	**Rs. 3,29,000** **(± 60,734)**
	$ 2,369	$ 1,391 (±712)	$ 142 (±84)	$ 73 (±61)	$ **3,948** (±**729)**

*Only those participants who had incurred indirect costs were considered

In all three public hospitals, medication costs formed the largest share of out-of-pocket expenditure except one public hospital (MAMC Delhi) that did not levy charges for procedures or investigations and provided a few subsidized drugs, resulting in significantly lower overall costs for patients. However, hormonal injections were not available at any of these sites. In comparison, the other public hospital in Chandigarh had a fixed hospital fee and additional costs for embryo freezing. Couples accessing another public hospital, SAT Trivandrum incurred the highest OOPE for medicines, cryopreservation and investigation.

In private hospitals, both had significantly higher fixed charges paid to the hospital compared to public hospitals, and similar to most public setups, medications accounted for the largest portion of expenses followed by expenditure for investigations. Embryo freezing and other ancillary services, like semen freezing and consultations, also contributed to the additional costs in both settings. At the private facility in Chennai, procedures like hysterolaproscopy, sonosalpingography, and cryopreservation were relatively charged higher than other sites, thus raising the overall procedure cost. Overall, both private hospitals followed a similar cost pattern of high fixed fees bundled with additional expenses for procedures and basic services, followed by considerable variable expenses primarily for medications and investigations.

**Fig 1:**
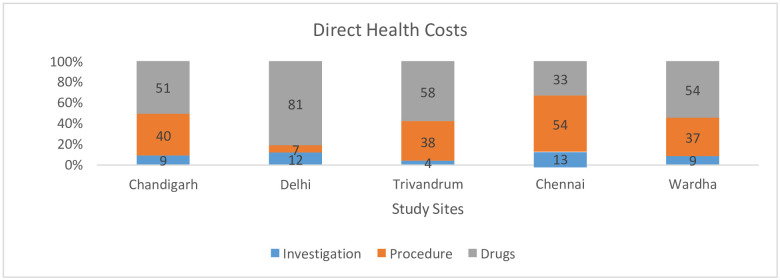
Direct Health Costs.

The share of different components in the total direct health costs, non-medical costs and indirect costs is depicted in Fig 1, highlighting that the expenses towards drugs were the highest.

**Fig 2:**
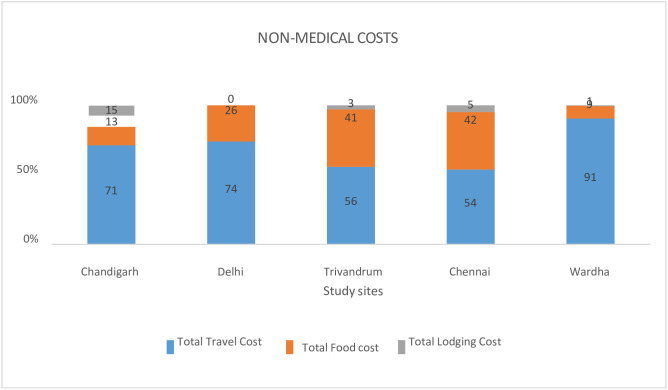
Non-Medical Costs.

As seen in Fig 2, participants do travel quite a distance to access care, and participants from three sites reported spending the highest amount on travel, as the median distance travelled from place of residence to the hospital was substantially more. More than 40% of the expenditure on non-medical costs was due to expenditure on food for the woman and her accompanying family member highest being noted in the southern sites. Expenses were also incurred towards the stay highest being at Chandigarh.

Table 3 describes the distance from residents’ homes to the IVF center. Participants from Wardha travelled a median distance of 119.7 km to reach the IVF center, while those from Chandigarh travelled 75.6 km. In contrast, participants from Chennai travelled the shortest median distance of 10 km. With a median travel distance of 43.9 km, participants from Trivandrum incurred the highest non-medical expenses. The public mode of transport reduced the indirect costs, although distance was greater.

**Table 3:** Distance from residence travelled by participants (IVF).

Study sites	Distance travelled in kms	
	Median	IQR (25^th^,75^th^)
**Chennai (Private)**	10	4,14.25
**Trivandrum (Public)**	43.9	20.5,57.5
**Delhi (Public)**	15	8.85,20
**Chandigarh** **(Public)**	75.6	31,96.25
**Wardha** **(Private)**	119.7	16,192.5

**Fig 3:**
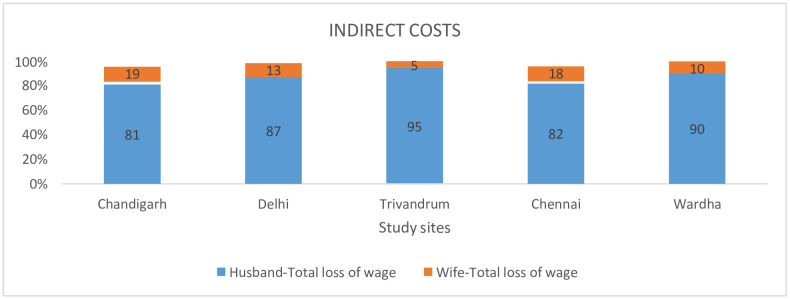
Indirect Costs.

Fig 3 reveals that loss of wages due to missed work of the male participants, accounted for more than 80% of indirect costs across all sites. Loss of wages of employed female participants was highest at Chandigarh and Chennai, followed by Delhi.

**Fig 4:**
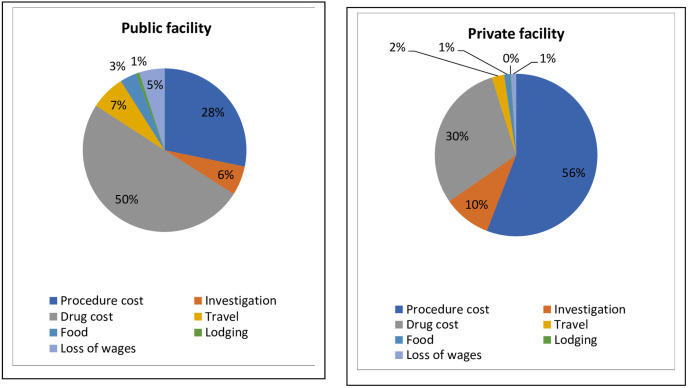
Share of components of OOPE on IVF.

Share of components of OOPE on IVF based on the type of facility. The overall average OOPE incurred for IVF was Rs. 1,61,893 (±1,08,254) [~USD 1943 (±1299)]. For those who availed service from the public facility, the average OOPE was Rs. 1,10,104 (±75,503) [~USD 1321 (±906)], and for those at the private facility, the average OOPE was Rs. 2,30,668(±1,09,556) [~USD 2,768 (±1315)]. Fig 4 portrays the various components of OOPE at public facilities; expenditure being highest for drugs, followed by procedure cost and investigation. At private facilities, the expenditure is highest for procedure costs, followed by drugs and investigation.

**Fig 5:**
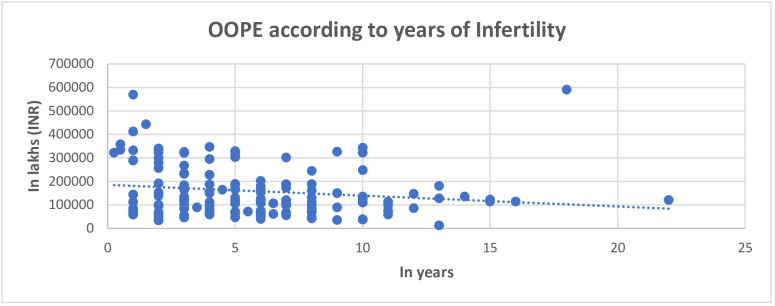
OOPE according to years of infertility.

Fig 5, indicates Out-of-pocket expenses (OOPE) were negatively correlated with the duration of infertility treatment, suggesting that OOPE incurred is higher in the early years of treatment.

OOPE was highest among older couples (41 + years), participants attending private facilities, those in higher income quintiles, and uninsured participants, reflecting substantial variation in financial burden across groups (Table 4). Other factors, including type of infertility, family size, and duration of infertility treatment, also contributed to differences in OOPE, underscoring the multiple determinants of IVF-related economic impact on households.

**Table 4:** Distribution of OOPE for IVF treatment by socio-demographic and clinical characteristics of participants.

Characteristics	Category	N (%)	Mean OOPE (USD)	Median OOPE (USD)
Average age of the couple	≤30	22 (14.9%)	145,563 (1,747)	83,294 (1,000)
	31–35	67 (45.3%)	167,213 (2,007)	120,861 (1,450)
	36–40	39 (26.4%)	142,262 (1,707)	118,265 (1,419)
	41+	20 (13.5%)	178,773 (2,145)	135,860 (1,630)
Family size	≤5	127 (85.8%)	164,529 (1,974)	124,215 (1,491)
	>5	21 (14.2%)	125,433 (1,505)	89,486 (1,074)
Income Quintile	Poorest	43 (29.1%)	124,879 (1,499)	113,369 (1,360)
	Poor	16 (10.8%)	132,436 (1,589)	112,977 (1,356)
	Moderate	31 (21.0%)	151,250 (1,815)	130,937 (1,571)
	Rich	33 (22.3%)	158,731 (1,905)	114,161 (1,370)
	Richest	25 (16.9%)	244,546 (2,935)	289,922 (3,479)
Insurance status	Uninsured	140 (95%)	151,290 (1,815)	120,318 (1,444)
	Insured	8 (5%)	293,580 (3,523)	300,801 (3,610)
Type of infertility	Primary	88 (59.5%)	161,739 (1,941)	125,550 (1,507)
	Secondary	60 (40.5%)	154,938 (1,859)	118,836 (1,426)
Facility type	Public	88 (59.5%)	110,105 (1,321)	89,060 (1,069)
	Private	60 (40.5%)	230,668 (2,768)	212,137 (2,546)
Infertility treatment	5 yrs or less	79 (53.3%)	185,210 (2,223)	153,602 (1,843)
	More than 5 yrs	69 (46.7%)	128,952 (1,547)	113,369 (1,360)

A multiple linear regression was conducted to identify predictors of out-of-pocket expenditure (OOPE) for infertility treatment. The model was statistically significant (p < 0.001) and explained 39.3% of the variance in costs (Adjusted R² = 0.393). Facility type was the strongest predictor (p < 0.001), with treatment at private facilities associated with an additional expenditure of Rs. 95,715 (66,007−125,423) compared to public facilities. Infertility treatment duration lesser than five years was significant (p = 0.002) associated with higher OOPE by ₹49,933 (18,960–80,906). Out-of-pocket expenditure increased significantly (p = 0.008) by Rs. 13,984 (3,717−24,251) for each higher income quintile. Factors such as age of the couple, number of family members and type of infertility) were not found to be significant predictors.

Assessment of determinants of CHE revealed that the single most significant determinant was the facility from which couples received treatment. As seen in Table 5, participants undergoing treatment at private facilities had 7.7 times higher odds of experiencing CHE compared to public facilities, which is statistically significant. Although primary infertility and lack of insurance coverage were associated with lower and higher odds of experiencing catastrophic health expenditure, respectively, this association was not statistically significant The model fit was statistically significant (p=<0.001), R2 = 0.449, and hosmer & lemeshow test p = 0.461.

**Table 5:** Determinants of Catastrophic Health Expenditure.

Factors associated with Catastrophic Health Expenditure	Catastrophic Health Expenditure OR (95% CI)	*P*
Facility type		
Public	1	<0.001
Private	7.727 (2.5-23.15)	
Type of infertility		
Secondary	1	0.088
Primary	0.41 (0.14-1.14)	
Insurance coverage		
Yes	1	0.74
No	1.38 (0.19-10.3)	
Financial support obtained		
No	1	0.005
Yes	4.54 (1.56-13.2)	
Number of infertility clinic treatments taken (median)		
Less than 3,	1	0.503
more than 3	1.42 (0.50-4.04)	
Years of undergoing infertility treatments (median)		
5 yrs or less,	1	0.10
more than 5 yrs	0.161 (0.40-0.65)	
Sperm donor used		
No	1	0.08
Yes	0.20 (0.03-1.26)	
Median annual family income		
Rs. 405000 & less, (USD 4860)	1	0.779
More than Rs. 405000	0.850 (.274-2.63)	
Median per capita income		
More than Rs. 120000 (USD 1440)	1	0.278
Rs. 120000 & less	1.929 (0.58-6.31)	
Median distance from residence		
25kms & less	1	0.870
more than 25 kms	1.085 (0.410-2.86)	
Cause of infertility		
Other factors	1	0.291
Tubal	0.576 (0.207-1.60)	
Male with risk factors		
No	1	0.031
Yes	0.238 (0.06-0.87)	
Female with risk factors		
No	1	0.348
Yes	1.60 (0.59-4.31)	
Married since (Median years)		
8 yrs or less	1	0.541
more than 8 yrs(1)	1.52(0.39-5.92)	
Male age (median years)		
36 yrs or below	1	0.233
Above 36 yrs	2.10 (0.62-7.13)	
Female age (median years)		
32 yrs or below	1	0.242
Above 32 yrs	0.499 (0.156-1.60)	

## Discussion

This study offers an insight into the out-of-pocket expenses incurred by couples undergoing IVF treatment at tertiary care facilities in India. It also gives a broad perspective on the most common causes of infertility among males and females, leading to IVF and the key components of costs and catastrophic health expenditure incurred by couples utilizing care in both public and private settings.

Majority (72%) of participants in our study undergoing IVF had female factor infertility.Tubal factor infertility was the leading cause among the female factors, as has been reported in Indian settings to affect nearly 10% − 25% of infertility [20]. Male infertility accounted to less than a qaurter (14%) also similar to other studies in India [21].

The findings of this study highlight the significant financial burden associated with in vitro fertilization (IVF) treatment, particularly for participants seeking care at private hospitals in India. The average out-of-pocket expenditure (OOPE) for IVF treatment was Rs. 1,10,104 (±75,503) [USD 1321 (± 906)] in public hospitals and Rs. 2,30,668(±1,09,556) [USD 2,768 (±1315)] in private hospitals, reflecting a clear disparity in costs depending on the type of facility. Having very few public facilities in India offering IVF, coupled with almost double the expenditure to avail services in private settings, exerts a tremendous financial burden on families, leading to CHE. Compared to spontaneous conceptions, IVF pregnancies also carry an elevated risk of obstetric complications, which require additional medical interventions related to antenatal care, delivery, and neonatal care resulting in prolonged hospital stays, contributing substantially to increased healthcare costs and unanticipated out-of-pocket expenses [22]. In India’s private sector, there is no regulatory cap on the cost of IVF, and the government has not introduced measures to control pricing. As a result, the cost can vary widely, often making IVF treatments unaffordable for many households, especially considering the country’s relatively low per capita income.

A systematic landscape analysis on IVF in Low and Middle-Income countries in, the South Asia region presents the typical cost of a single IVF cycle being USD 2500 and above, which is nearly similar to the cost per cycle found in our study [23]. This estimate is considerably lower than the expenditures incurred by couples in developed countries like the USA [24]. According to estimates from the U.S. Department of Health and Human Services in 2024, the cost of a single IVF cycle typically ranges between US$15,000 and US$20,000, and may exceed US$30,000 when donor eggs or additional services are involved [25].

As per the survey from National Sample Survey Office (NSSO) in India, high OOP health expenditures can drive many households to poverty, as the poverty headcount in the population rose to 19.05% from 16.44%, after these payments. This 2.61% increase in the poverty rate represented an additional 6.47 million households [26]. Similarly, a study conducted in South Africa suggested that 51% of the poorest study participants & one in five couples in total faced catastrophic out-of-pocket health expenditure for assisted reproductive technique with conventional ovarian stimulation [27]. Beyond the standard planned stages, IVF is associated with risks such as ovarian hyperstimulation syndrome (OHSS), which can substantially elevate out-of-pocket expenditure. Managing OHSS may involve hospitalization, close clinical monitoring, and additional medication further adding to the financial burden. These complications not only increase costs but may also impact clinical outcomes, highlighting that the economic implications of IVF extend beyond routine procedures to encompass the cost of managing adverse events [28].

A negative correlation was observed between relative ART costs and GDP per capita, with African and South-East Asian countries reporting ART costs that were, on average, up to 200% of their GDP per capita. In contrast, regions such as the Americas and the Eastern Mediterranean experienced relatively lower ART costs, which were often associated with the presence of regulatory frameworks and government funding mechanisms for fertility treatments [17].

In India, where the GDP per capita is approximately USD 2,698, the average annual income is about INR 384,000 (~ USD 4,554). Our study found that the median income of participants was INR 360,000, (~$4320) with a range from INR 240,000 to INR 600,000 (~$2880 to $7200). The cost of an IVF cycle in India ranges from INR 1 lakh to 2.5 lakh, which represents 28% to 69% of the median annual income for IVF treatment resulting in significant financial burden.. These findings highlight the need for greater access to affordable services within government set ups and insurance coverage to benefit a larger segment of the population.

Several countries, such as France, Germany, and the United Arab Emirates, provide free or subsidized IVF services through their national healthcare systems. For example, the Netherlands reimburses up to three IVF cycles, and the UK offers free IVF treatment within specific age limits. In the U.S., the Affordable Care Act aims to include reproductive technologies in essential health benefits, which could improve access to IVF. While there are concerns about resource allocation for couples with low chances of conception, wealthier countries with declining birth rates have expanded IVF coverage. In contrast, many developing nations, particularly in Asia and Africa, struggle to provide affordable IVF due to the perception that infertility is a less urgent issue in overpopulated regions. Nevertheless, affordable IVF options, such as the $300 package offered in Sudan and Tanzania, demonstrate that low-cost solutions can increase access, giving more couples the opportunity to pursue IVF and achieve pregnancy [29].

In 2012, the Indian Central Government Health Scheme (CGHS) provided reimbursement to its beneficiaries of up to INR 65,000 (~$ 780) for up to three IVF cycles; this amount only covers the cost of drugs, disposables, and monitoring [30]. It does not include other major expenses such as the IVF procedure itself, laboratory fees, or the cost of the embryo transfer. As a result, beneficiaries of this scheme would yet face significant out-of-pocket expenses beyond the reimbursement for a comprehensive IVF treatment.

With the introduction of Ayushman Bharat Pradhan Mantri Jan Arogya Abhiyan (PM-JAY), it is envisaged that primary health care access is improved and out-of-pocket expenditure is reduced. A study conducted on the PMJAY suggested the beneficiaries had a higher probability of visiting a private PMJAY empaneled hospital for any ailment and reported a decrease in the Out-of-pocket expenditure. This reduced the immediate financial burden of enrolled households [31].

However, the PMJAY scheme is limited to inpatient hospital care-related expenses and expenses for medicines form over 70% of the total out-of-pocket expenditure [32]. Our study found similar results, showing that most direct medical healthcare expenditures were on medicines. Causes for infertility, such as PCOS, need multidisciplinary management apart from the increasing cost of medicines. These clinics need to be established at public healthcare systems in India to manage the comorbidities existing alongside PCOS that can affect the outcomes of the pregnancies [33]. IVF and other fertility treatments are not included under the current list of services covered by PMJAY. However, some state government schemes may offer subsidized fertility treatment options at select public hospitals, but these are often limited and do not cover the entire cost of IVF. Fertility treatments are generally not included in most standard health insurance plans, leaving many participants to rely on out-of-pocket payments. Thus, including all components of IVF in the insurance plan if being considered would be essential as most of the procedures are day care or OPD based.

Although India has reached a replacement fertility level below 2, in sharp contrast, infertility is equally on the rise. The primary health care is oblivious to this in terms of providing counselling, screening, and basic management of associated factors.

To summarize, the data demonstrates an urgent need for policy intervention to improve the affordable access to infertility services in India, including IVF, that could ensure financial risk protection to those vulnerable to impoverishment due to infertility.

### Strengths

Infertility and access to IVF are rising public health challenges in India impoverishing many households, making this study highly important. Primary data from couples seeking services from both public and private settings gives a perspective of the vast differences in expenditure incurred. The study is relevant for both social and policy considerations and presents recommendations to expand insurance coverage, enhancing public healthcare infrastructure, and promoting equitable access to infertility services, offering valuable guidance for policymakers.

### Limitations

The estimation of out-of-pocket expenditure (OOPE) was based on participants’ self-reported data, which may be influenced by recall bias although minimal.

The study was conducted in selected tertiary care hospitals (two private and three public) at selected sites in India which may limit the generalizability of the findings to other healthcare settings.

## Supporting information

S1 File Structured questionnaire. (PDF)
