# Supplementary material for: Out-of-pocket expenditure experienced by couples seeking In Vitro Fertilization (IVF) services at tertiary care facilities in India
Source: PLoS One. 2026 Jul 15;21(7):e0351080. doi: 10.1371/journal.pone.0351080 (PMC13372168; doi:10.1371/journal.pone.0351080)
Supplement: S1 File — (PDF) [file pone.0351080.s001.pdf]

To estimate cost of diagnosis of infertility and its management including In Vitro Fertilization (IVF) and Quality of Life among infertile couples

Data collection for participants availing IVF services

General information sheet

Date: ____________

Enrolment number: ☐ ☐ ☐ ☐

Section 1: General Information

**Variables**	**Male**	**Female**
Name		
Age (in years)		
Address		
Phone number		
Education		
Occupation		
**Variables**	**For the couple**	
Number of members in household		
Total monthly income of the participant	Male:<br><br>Female:	
Total monthly income of family (including salary, rent etc)		

Section 2: Household consumption expenditure

How much does your family spend per month on following items:	Expense		
	7 days	30 days	365 days
1. Food: ration (Cereals, pulses, edible oil, bread etc.), Fruits and vegetables, Milk, Milk products, Beverages etc			
2. Education (Books, newspaper, fees)			
3. Health (including infertility and diagnosis)			
4. Infertility diagnosis and treatment			
5. Bills (Electricity, telephone, water, internet)			
6. Conveyance, fuel			
7. Rents			
8. Clothing, Footwear, bedding, curtains etc			
9. Entertainment (Cable, cinema, sports, travel, vacation, recreation & hobbies)			
10. Personal items (Watch, mobile phone, spectacles, toiletries, jewelry, fitness, any medical equipment's like hearing aids etc…))			
11. Consumer services (Domestic help, cook, sweeper, barber, tailor, priest, beautician, personal grooming)			
12. Pan, Tobacco, alcohol or any other intoxicants			
13. Miscellaneous (household appliances, home maintenance, furniture, crockery, pets, or any family function, charity donations, gifts, professional services eg finance advice, lawyer etc…)			

14. Savings (Insurance, retirement, investments etc)			
15. Total Expenditure<br>[Total to be calculated by the coordinating institute]			

Section 3: Insurance details

Do you or your partner have any insurance coverage for this treatment?	1. Yes<br>2. No
If Yes,<br><br>What is the name of the insurance?	
How much do you pay instalment for the insurance in a year?	
How much of your total treatment expense was covered by the insurance?	
Any other financial support obtained?	1. Yes<br>2. No
If Yes,<br><br>What was the source of finance for all the expenditure incurred on infertility diagnosis and treatment	1. Incomes/ Savings<br>2. Borrowing (IF yes, mention the source of borrowing)<br><br><br>3. Sale of asset (IF yes, what was the asset?)<br><br><br>4. Contributions from family and friends<br>5. Insurance<br>6. Others (If yes, kindly mention)

Section 4: Case history

Married since (in years)	
Undergoing infertility treatment since (in years)	

Number of infertility clinics/ centers sort treatment from so far		
	Male	Female
Presence of infertility factor	1. Azoospermia<br>2. Oligozoospermia<br>3. Ashtenospermia<br>4. Teratozoospermia<br>5. Obstruction<br>6. Varicocoele<br>7. Overweight/ Obese<br>8. Ejaculatory dysfunction<br>9. Malignancy (current/ past treatment of)<br>10. Unexplained<br>11. Others	1. Ovarian<br>2. Tubal<br>3. Uterine<br>4. PCOS<br>5. Tuberculosis<br>6. Endometriosis<br>7. Fibroids<br>8. Unexplained<br>9. Others
Family history of infertility	1. Yes<br>2. No	1. Yes<br>2. No
Tobacco consumption	1. Yes<br>2. No	1. Yes<br>2. No
Alcohol consumption	1. Yes<br>2. No	1. Yes<br>2. No
Type of infertility	1. Primary<br>2. Secondary	
Past obstetric history	Gravida<br><br>Para<br><br>Number of living children:<br><br>Number of abortions:<br>1. Spontaneous<br>2. Induced	
Have you undergone any IUI before?	1. Yes<br>2. No	
If yes, how many times was IUI done?		
Is any donor sperm being used in the current cycle?	1. Yes<br>2. No	

Section 5a: Current health status (Female)

**Parameters**		If yes,<br>Name of drugs	If yes,<br>Cost of drugs/ month	If yes,<br>Duration of treatment
Diabetes	1. Yes<br>2. No			
Hypertension	1. Yes<br>2. No			
Any other illness, specify				

**Variables**	**Response**
Have you undergone any surgeries before?	1. Yes<br>2. No
If yes, give details here	

Section 5b: Current health status (Male)

**Parameters**	Tick if on treatment	Name of drugs	Cost of drugs/ month	Duration of treatment
Diabetes	1. Yes<br>2. No			
Hypertension	1. Yes<br>2. No			
Any other illness, specify				

**Variables**	**Response**
Have you undergone any surgeries before?	1. Yes<br>2. No
If yes, give details here	

Section 7: Questionnaire for out of pocket expenditure

Section 7a: IVF/ ICSI

Variables	Response
Was a package cost paid at the hospital for the IVF/ ICSI procedure?	1. Yes<br>2. No
If yes, How much was the paid package cost?	
Was any additional costs paid other than the package cost for any of the procedures?	1. Yes<br>2. No
Is yes, what was the amount paid?	

Section 7b: Pre-IVF Cycle

If costs paid from the package costs mark P (mention the breakup of the package cost below)

Direct health costs									
**Variables**	**1st cycle**			**2nd cycle**			**3rd cycle**		
	**Unit cost**	**Number of visits**	**Total cost**	**Unit cost**	**Number of visits**	**Total costs**	**Unit cost**	**Number of visits**	**Total costs**
Registration									
Consultations									
Blood tests									

ECG<br>X ray<br>HSG<br><br><br>Scortal Doppler (male)									
USG									
Scopy<br>Hysteroscopy<br>Laparoscoy<br>Sonosalpingohysterosgr aphy									
Drugs<br>Inj HMG<br>Inj FSH<br>Letrozole<br>Clomiphene citrate (CC)									

<table>
<tr><td>Others</td><td></td><td></td><td></td><td></td><td></td><td></td><td></td><td></td><td></td></tr>
<tr><td colspan="10"><u>Non-Medical costs</u></td></tr>
<tr><td>Distance from residence</td><td colspan="3"></td><td colspan="3"></td><td colspan="3"></td></tr>
<tr><td>Travel time</td><td colspan="3"></td><td colspan="3"></td><td colspan="3"></td></tr>
<tr><td>No of visits (with dates)</td><td colspan="3"></td><td colspan="3"></td><td colspan="3"></td></tr>
<tr><td>Travel cost per visit (including bystander)</td><td colspan="3"></td><td colspan="3"></td><td colspan="3"></td></tr>
</table>

Total travel cost (including bystander) [Total to be calculated by the coordinating institute]			
Food cost per visit (including bystander)			
Total food cost [Total to be calculated by the coordinating institute]			
Lodging/ boarding cost per visit (including bystander)			
Total Lodging/ boarding cost [Total to be calculated by the coordinating institute]			
Other overhead payments per visit (including bystander)			

Total overhead payments (including bystander)<br>[Total to be calculated by the coordinating institute]			
Informal payments per visit			
Total informal payments<br>[Total to be calculated by the coordinating institute]			
Other payments			
Total costs<br>[Total to be calculated by the coordinating institute]			

**Indirect costs**			
Did you miss any wages during the hospital visit? If yes, answer the following questions. If No, mark No and move to the next section.<br>1. Yes<br>2. No			
**Wife**			
Days of work missed			
Amount of wage missed wage for the day			
Average monthly wage			
Total loss of wage [Total to be calculated by the coordinating institute]			
**Husband**			
Days of work missed			
Amount of wage missed wage for the day			
Average monthly wage			
Total loss of wage [Total to be calculated by the coordinating institute]			
Other accompanying person			
Days of work missed			

Amount of wage missed wage for the day			
Average monthly wage			
Total loss of wage [Total to be calculated by the coordinating institute]			
**Any other additional information**			

<u>Section 7c: Additional expenses in between the cycles (if any)</u>

Was any other expenses incurred in between the cycles? If yes, mention the scenario below and give	Drugs	Investigations	Procedure	Indirect medical costs	Loss of wage

**details below the relevant column.**					
Mention in between which cycle					

Section 7d: Follicle study

If costs paid from the package costs mark P (mention the breakup of the package cost below)

Direct health costs									
**Variables**	**1st cycle**			**2nd cycle**			**3rd cycle**		
	**Unit cost**	**Number of visits**	**Total cost**	**Unit cost**	**Number of visits**	**Total costs**	**Unit cost**	**Number of visits**	**Total costs**
Registration									
Visits									
Blood tests<br>Serum Estradiol (E2)<br>Serum Progesterone (P)<br>Others									
USG									
Drugs									

Trigger HCG<br>Trigger Antogonist<br>Others									

**Non-Medical costs (Total to be calculated by the coordinating institute)**			
Distance from residence			
Travel time			
No of visits (with dates)			
Travel cost per visit (including bystander)			

Total travel cost (including bystander)			
Food cost per visit (including bystander)			
Total food cost			
Lodging/ boarding cost per visit (including bystander)			
Total Lodging/ boarding cost			
Other overhead payments per visit (including bystander)			
Total overhead payments (including bystander)			

Informal payments per visit			
Total informal payments			
Other payments			
Total costs			
**Indirect costs**			
Did you miss any wages during the hospital visit? If yes, answer the following questions. If No, mark No and move to the next section.<br>1. Yes<br>2. No			
**Wife**			
Days of work missed			
Amount of wage missed wage for the day			
Average monthly wage			
Total loss of wage			

**Husband**			
Days of work missed			
Amount of wage missed wage for the day			
Average monthly wage			
Total loss of wage			
Other accompanying person			
Days of work missed			
Amount of wage missed wage for the day			
Average monthly wage			
Total loss of wage			
**Any other additional information**			

Section 7e: Oocyte retrieval/ Ovum pickup

If costs paid from the package costs mark P (mention the breakup of the package cost below)

Direct health costs									
**Variables**	**1st cycle**			**2nd cycle**			**3rd cycle**		
	**Unit cost for the procedure**	**Number of visits**	**Total cost**	**Unit cost for the procedure**	**Number of visits**	**Total costs**	**Unit cost for the procedure**	**Number of visits**	**Total costs**
Registration									
Consultations									

Procedure cost									
Drugs									
Any overhead charges									
Additional costs for semen donation									
Costs paid for donor sperm (if applicable)									
**<u>Non-Medical costs</u>**									
Distance from residence									
Travel time									
No of visits (with dates)									

Travel cost per visit (including bystander)			
Total travel cost (including bystander) [Total to be calculated by the coordinating institute]			
Food cost per visit (including bystander)			
Total food cost [Total to be calculated by the coordinating institute]			
Lodging/ boarding cost per visit (including bystander)			

Total Lodging/ boarding cost [Total to be calculated by the coordinating institute]			
Other overhead payments per visit (including bystander)			
Total overhead payments (including bystander) [Total to be calculated by the coordinating institute]			
Informal payments per visit			
Total informal payments [Total to be calculated by the coordinating institute]			
Other payments			

Total costs<br>[Total to be calculated by the coordinating institute]			
**<u>Indirect costs</u>**			
Did you miss any wages during the hospital visit? If yes, answer the following questions. If No, mark No and move to the next section.<br>1. Yes<br>2. No			
**Wife**			
Days of work missed			
Amount of wage missed wage for the day			
Average monthly wage			
Total loss of wage<br>[Total to be calculated by the coordinating institute]			
**Husband**			
Days of work missed			
Amount of wage missed wage for the day			
Average monthly wage			
Total loss of wage			

[Total to be calculated by the coordinating institute]			
Other accompanying person			
Days of work missed			
Amount of wage missed wage for the day			
Average monthly wage			
Total loss of wage [Total to be calculated by the coordinating institute]			
**Any other additional information**			

Section 7f: Complications

If costs paid from the package costs mark P (mention the breakup of the package cost below)

Did you have any episode of OHSS	1. Yes<br>2. No
If yes,<br>Was there any hospitalization?	1. Yes<br>2. No
If yes,<br>For how many days were you hospitalized?	
What were the costs for hospital admission?	
What were the costs for drugs?	
Any other costs were paid in relation to OHSS?	

Did you have any other complications?	1. Yes<br><br>2. No
If yes,<br><br>Provide details of all costs paid for the same	

	**Non-Medical costs**
Distance from residence	
Travel time	
No of visits (with dates)	
Travel cost per visit (including bystander)	

Total travel cost (including bystander) [Total to be calculated by the coordinating institute]	
Food cost per visit (including bystander)	
Total food cost [Total to be calculated by the coordinating institute]	
Lodging/ boarding cost per visit (including bystander)	
Total Lodging/ boarding cost	

[Total to be calculated by the coordinating institute]	
Other overhead payments per visit (including bystander)	
Total overhead payments (including bystander) [Total to be calculated by the coordinating institute]	
Informal payments per visit	
Total informal payments [Total to be calculated by the coordinating institute]	
Other payments	

Total costs [Total to be calculated by the coordinating institute]	
**<u>Indirect costs</u>**	
Did you miss any wages during the hospital visit? If yes, answer the following questions. If No, mark No and move to the next section.<br>1. Yes<br>2. No	
**Wife**	
Days of work missed	
Amount of wage missed wage for the day	
Average monthly wage	
Total loss of wage [Total to be calculated by the coordinating institute]	
**Husband**	
Days of work missed	
Amount of wage missed wage for the day	
Average monthly wage	
Total loss of wage	

[Total to be calculated by the coordinating institute]	
**Other accompanying person**	
Days of work missed	
Amount of wage missed wage for the day	
Average monthly wage	
Total loss of wage [Total to be calculated by the coordinating institute]	
**Any other additional information**	

Section 7g: Embryo transfer (ET) and Frozen Embryo transfer

If costs paid from the package costs mark P (mention the breakup of the package cost below)

**Direct health costs**									
**Variables**	**1st cycle**			**2nd cycle**			**3rd cycle**		
	Unit cost for the procedure	Number of visits	Total cost	Unit cost for the procedure	Number of visits	Total costs	Unit cost for the procedure	Number of visits	Total costs
Registration									
Consultations									

Blood tests<br>FS<br>Serum estradiol (E2)<br>Serum Progesterone (P)<br>Others									
Procedure cost									
Drugs<br>Estrogen tablets<br>Progesterone<br>Beta HCG (for 1 days)<br>Others									

Additional costs for cryopreservation of extra embryos									
**Non-Medical costs**									
Distance from residence									
Travel time									
No of visits (with dates)									
Travel cost per visit (including bystander)									

Total travel cost (including bystander) [Total to be calculated by the coordinating institute]			
Food cost per visit (including bystander)			
Total food cost [Total to be calculated by the coordinating institute]			
Lodging/ boarding cost per visit (including bystander)			
Total Lodging/ boarding cost [Total to be calculated by the coordinating institute]			

Other overhead payments per visit (including bystander)			
Total overhead payments (including bystander) [Total to be calculated by the coordinating institute]			
Informal payments per visit			
Total informal payments [Total to be calculated by the coordinating institute]			
Other payments			

Total costs [Total to be calculated by the coordinating institute]			
**Indirect costs**			
Did you miss any wages during the hospital visit? If yes, answer the following questions. If No, mark No and move to the next section.<br>1 Yes<br>2 No			
**Wife**			
Days of work missed			
Amount of wage missed wage for the day			
Average monthly wage			
Total loss of wage			
**Husband**			
Days of work missed			
Amount of wage missed wage for the day			
Average monthly wage			
Total loss of wage [Total to be calculated by the coordinating institute]			
**Other accompanying person**			

Days of work missed			
Amount of wage missed wage for the day			
Average monthly wage			
Total loss of wage [Total to be calculated by the coordinating institute]			
**Any other additional information**			

Section 7h: Additional costs

Cost for TESE/ TESA (if applicable)	
Was the costs paid from the package costs? (If yes, mark P)	**1.** P 2. N
**Non-Medical costs**	
Distance from residence	
Travel time	
Travel cost per visit (including bystander)	
Total travel cost (including bystander)	
Food (including bystander)	
Lodging/ boarding (including bystander)	
Other overhead payments (including bystander)	
Informal payments	
Other payments	
Total costs<br>[Total to be calculated by the coordinating institute]	
**Indirect costs**	
Did you miss any wages during the hospital visit? If yes, answer the following questions. If No, mark No and move to the next section.<br>1. Yes<br>2. No	
**Wife**	
Days of work missed	
Amount of wage missed wage for the day	

Average monthly wage	
Total loss of wage<br>[Total to be calculated by the coordinating institute]	
**Husband**	
Days of work missed	
Amount of wage missed wage for the day	
Average monthly wage	
Total loss of wage<br>[Total to be calculated by the coordinating institute]	
**Other accompanying person**	
Days of work missed	
Amount of wage missed wage for the day	
Average monthly wage	
Total loss of wage<br>[Total to be calculated by the coordinating institute]	
**Any other additional information**	

Section 8: Additional remarks (if any)

In case the patient reports any other **additional remarks** related to cost of the last 3 cycles within the past 2 years, mention in the table below
